# Some Methods of Preparing Teeth for Histological Examination

**Published:** 1903-01

**Authors:** 


### Reviews of Dental Literature.

Some Methods of preparing Teeth for Histological Examination. By A. P. Beddard, M.D, and E. I. Spriggs, M.D., Gull Research Student.[1]

The object of this short paper is to lay before the Society our experience with certain histological methods, and especially those suitable for class purposes. Our attention has been mainly directed towards trying to find the best method of preparing sections of the hard and soft tissues together by decalcification, for it is clear that any method of grinding down the hard and soft tissues together entails not only great labor and waste of material, but also the fundamental objection that most of the sections so obtained are, from a histological point of view, inordinately thick. Every one who has to prepare a large number of sections for a class knows that it is easy by almost any method to produce an occasional good lot of sections, but when a large number of satisfactory sections are wanted the methods adopted require careful selection. The chief method, then, we wish to mention is the one we have used for two years to prepare sections of the hard and soft tissues together by decalcification.

The whole problem of decalcification, whether it be of bone, corals, sponges, or teeth, is to find a decalcifying fluid of such a nature that, after proper fixation of the tissues, it will abstract the lime salts without doing any damage to the soft tissues. In choosing a decalcifying fluid, therefore, it is necessary to have some criterion of whether the soft tissues have or have not been injured. In extreme cases of damage by acids the soft tissues may refuse to stain at all. The most beautiful looking section of a decalcified tooth we have seen was produced by decalcifying with strong nitric acid and phloroglucin; decalcification was complete in half an hour. Unstained the sections looked nearly perfect, but they obstinately refused to stain. This is an extreme case.

The real criterion of whether the soft tissue has been injured during decalcification is the condition of the nucleus as seen when

[1] Demonstrators of Physiology and Dental Histology, Guy's Hospital.

stained by a basic stain, such as hæmatoxylin. If uninjured a normal, darkly stained nuclear net-work is seen within the nucleus; when injured to the slightest extent no such nuclear net-work is visible, the nucleus staining a uniform blue color.

Taking this as the criterion of a decalcifying fluid, we have tried a number of the published formulæ and have found, as others have done, that mineral acids in water, although rapid in action, damage the soft tissues to a greater or less extent. Numerous formulæ containing additions of phloroglucin, alum, or sodium chloride have been suggested in order to counteract this action of the acid, but we have found them to give most uncertain results.

The fluid which we have found most satisfactory is a mixture of nitric acid in alcohol. This fluid, if properly used, gives uniformly good results. The method we have adopted is to saw the tooth in half and then thoroughly fix it in either perchloride of mercury or formalin. With perchloride of mercury we have used a saturated watery solution acidified with one per cent. glacial acetic acid. In this it remains three days. The mercury is thoroughly washed out in frequent changes of seventy per cent. spirit (which gives better results than washing out in water), and is then transferred to ninety-five per cent. spirit for one week.

With formalin we have used a ten per cent. solution in water. In this the tooth remains at least a week, but it may remain much longer without harm. The formalin is washed out in running water and the tooth is passed through seventy per cent. spirit for two days, to ninety-five per cent. spirit for one week. A thorough fixation and hardening with some fixative agent and alcohol is essential before decalcification. From the ninety-five per cent. spirit the tooth is transferred to a mixture of five per cent. white nitric acid in ninety-five per cent. spirit. This fluid was suggested and used by Mayer many years ago for the decalcification of bone. It has to be changed every day,—this is essential to its success,—and, further, it must be freshly prepared every time it is used, for the acid rapidly oxidizes the alcohol. At the end of about a week all the enamel will have been removed. The process is continued for another week; at the end of that time the tooth will be nearly decalcified, but the dentine remains so hard that it is difficult to cut it with a knife.

At the end of this fortnight the tooth is transferred to a mixture of three per cent. white nitric acid in seventy per cent. alcohol. The originator of this mixture is unknown; it has been used for the decalcification of bone, and we obtained the formula from Bolles Lee's "Microtomist's Vade Mecum." In this fluid the dentine rapidly softens, and as soon as a needle can be easily pushed through it decalcification is complete; this will be at the end of one or more days. As a matter of fact, it is unnecessary and much better not to push needles into the tooth to test how the decalcification is progressing. In this fluid it can be seen that the tooth becomes increasingly translucent from without inward, and a very little experience soon tells one at a glance when that process has been completed.

The next step is to get rid of the acid. Rapid neutralization of the acid by lithium carbonate or any other similar salt is certainly a mistake; the rapid liberation of bubbles of gas within the soft tissues is liable to tear and destroy fine fibrils. A better method is to place the pieces of tooth in running tap-water for a day; even better results are obtained by washing out the acid in frequent changes of seventy per cent. spirit until the fluid remains neutral to litmus.

Another excellent decalcifying fluid is thirty-three per cent. pure formic acid in water. We do not know who originally suggested it, but we obtained the formula from Dr. Römer, of Strassburg. Formic acid being itself a fixative agent of considerable power does not, even in strong solutions, tend to gelatinize and damage the tissues as do mineral acids in water. The results obtained by this fluid are quite as good as, if not slightly better than, those with nitric acid in alcohol, and in some ways this fluid is the more convenient, as it is more rapid, taking about fourteen days, and does not need to be changed more than once or twice. The acid is got rid of by placing the tooth in running tap-water for a day.

Embedding is the next process to be considered. There is a choice of three media,—paraffin, gum, and celloidin.

We have tried paraffin, and found that it is impossible to cut decalcified adult teeth in it, owing to the extreme hardness which the dentine assumes during the process of embedding.

Gum is the simplest medium, but it is uncertain and not wholly suitable for class purposes, for when thin sections of about ten microns in thickness of hard and soft tissues together are required, unless the embedding be perfect the hard tissues in the process of cutting tear away from the soft tissues. It is true that gum when frozen to exactly the right hardness is an excellent embedding medium and allows good sections to be cut, but it is difficult in practice to keep gum frozen to exactly the right point for a sufficient time to allow several hundred sections to be cut at once. If the gum be too hard, thin sections curl up badly; if too soft, the embedding is imperfect and the soft tissues are torn. Further, when the gum has been dissolved out the soft tissues are unsupported and easily torn by an inexperienced student in the process of staining and mounting.

Embedding in celloidin gets over all these difficulties, and the results obtained well compensate for the extra time and trouble.

We only touch upon the subject of cutting the bits of decalcified teeth embedded in celloidin in order to mention the Delépine freezing microtome to any who are unacquainted with its merits. It is only the excellence of this microtome which makes the cutting of sections in celloidin for a whole class possible. It is automatic, and enables sections of any desired thickness up to forty microns to be cut at a rate of several hundred in a few minutes,—in fact, enough to last any ordinary class for years.

In order to prepare the celloidin embedded material for cutting in gum it is necessary to get rid of the eighty per cent. spirit in which the celloidin has been hardened by placing the bits of teeth in running water for twenty-four hours. They are then placed for another day in a mixture of gum and syrup (Cole, quoted by Bolles Lee, *loc. cit.*):

> Gum mucilage, B.P., 5 parts;
> Syrup (1 lb. loaf sugar in Oi boiling water), 3 parts;
> Carbolic acid, gr. 5 to ℥i. of medium; and, just before cutting, a piece is taken out of this, wiped, and frozen in gum without sugar. For class purposes we cut all our sections ten microns thick, which is as thin as an ordinary paraffin section of a soft tissue. By this method and with this instrument, however, sections of only six microns thick can be cut when required, as for the study of bacteria in the hard and soft tissues.

When cut the sections are placed in water to dissolve out the gum, and then into bottles containing eighty per cent. spirit, in which they will keep indefinitely and can be given out as required.

There is only one other point with regard to these celloidin sections which we wish to mention, and we do so because until we found the solution of the difficulty it caused us much trouble. The point is that celloidin sections of teeth take a long time to dehydrate perfectly in absolute alcohol. If the student imperfectly dehydrates the section—and he may easily do it—and then places it into a clearing agent such as xylol, the section becomes permanently wrinkled and is spoilt. The same is true, though to a less extent, of other clearing agents, such as oil of cedar-wood or oil of bergamot. The solution of the difficulty is to be found in a clearing mixture suggested by Weigert, which consists of three parts xylol to one part of white anhydrous crystalline carbolic acid. The carbolic acid in the mixture will take up large quantities of water, and sections out of ordinary methylated spirit are almost instantly cleared without any danger of wrinkling. In using this fluid it is necessary to remember that it cannot be used for sections stained by basic aniline dyes such as fuchsin, methylene blue, and thionin blue, because it rapidly decolorizes them. When these basic dyes have been used, as in staining for bacteria, the sections are best cleared in aniline oil, and then in a mixture of one part of aniline oil in three parts of xylol.

Another method which we wish to mention is the one we have found most useful in preparing decalcified sections of the hard and soft tissues together, in order to demonstrate the distribution of bacteria in them. The difficulty has been to find a decalcifying fluid which does not in any way impair the staining properties of bacteria, and this, we think, we have found in trichloracetic acid. It is well known that bacteria after exposure to the action of mineral acids stain badly; and even the addition of alcohol to the acid does not wholly prevent this. A ten per cent. solution of trichloracetic acid in water has long been used in physiology as a precipitant of proteids; in other words, it is a fixative agent of considerable power, and when we found that its calcium salt was soluble, we tried it as a decalcifying agent. Judged as a general decalcifying agent by its effect upon the staining properties of the nuclei in the soft tissues, the results are slightly inferior to those obtained with either formic acid or nitric acid and alcohol. But from the point of view of the bacteria, trichloracetic acid leaves nothing to be desired.

The method we have adopted is as follows: The tooth is sawed across and fixed in perchloride of mercury or formalin, and hardened in alcohol just as before. It is then transferred to a five per cent. solution of trichloracetic acid in water. This is the best strength to use; damage to the soft tissues is minimal, and decalcification is sufficiently rapid. In about a week the tooth becomes almost as flexible as a piece of india-rubber, and decalcification is complete. The fluid has to be changed once or twice, but it need not be freshly prepared. The acid is washed out in running tap-water, and the teeth are embedded in celloidin, and set in gum as before.

In order to obtain the best results, the section should not be more than six microns thick; but even with a Delépine microtome it is difficult to cut a large number of whole sections as thin as this, and for class purposes a thickness of ten microns is sufficiently thin. Before staining, but after the gum has been washed out in water, it is well to place the sections in methylated spirit for some hours; for some reason this greatly improves the staining properties of the bacteria.

There are other methods which we might mention, but we think we shall have trespassed upon your patience sufficiently long. With regard to the sections under the microscopes, it is only fair to them and to the methods by which they have been produced to state that they are not specimens prepared with any special care. They are in all cases taken from the bottles of sections cut for a class, and therefore represent simply the average result which may be expected. We do not doubt that if special care were given to the preparation of any given specimen, these methods could yield still better results.—*Transactions of the Odontological Society of Great Britain.*

